# Unlocking the Potential: A Systematic Literature Review on the Impact of Donor Human Milk on Infant Health Outcomes

**DOI:** 10.7759/cureus.57440

**Published:** 2024-04-02

**Authors:** Vijiya Kashyap, Sonali G Choudhari

**Affiliations:** 1 Department of Community Medicine, Jawaharlal Nehru Medical College, School of Epidemiology and Public Health, Datta Meghe Institute of Higher Education and Research, Wardha, IND

**Keywords:** immunity, preterm, low birth weight, lactation management, human milk bank

## Abstract

Human mother milk is considered the most healthy and best source of nutrition for both premature and full-term infants, as it possesses many health benefits and is associated with its consumption. Some of the mothers are not able to produce an adequate quantity of milk to meet the required needs of the infants, particularly in cases involving premature births or facing challenges in breastfeeding. Especially for the most vulnerable premature infants, donor human milk (DHM) provides a helpful bridge for effective breastfeeding. Even with the advancement in baby formulas, no other dietary source can match the bioactive matrix of benefits found in human breast milk. This literature review discusses the risks associated with prematurity and explores the use of DHM in the care of premature infants. It helps prevent substantial preterm complications, especially necrotizing enterocolitis, bronchopulmonary dysplasia, and late-onset sepsis, which are more commonly seen in infants who are given formulated milk made from cow's milk. It gives insights into the benefits of DHM, such as immunological and nutritional benefits, which is a basic infant’s need. When medical distress prevents mothers from producing enough breast milk for their infants, pasteurized human donor breast milk should be made accessible as an alternative feeding option to ensure infants remain healthy and nourished. A systematic literature search was conducted using PubMed and Google Scholar databases and other sources. A total of 104 articles were searched, of which 35 were included after identification, filters were applied, eligibility was checked, and references out of scope were excluded. Human milk banking should be incorporated into programs encouraging breastfeeding, highlighting lactation in mothers and only using DHM when required.

## Introduction and background

Worldwide, merely 38% of infants meet the WHO’s recommendation to breastfeed exclusively for the initial 180 days (six months) of life, even though the guidelines support this practice [1]. While a mother's own milk is the preferred choice for all newborns, a significant proportion of many preterm and critically ill infants may not receive an adequate amount of breast milk during the initial days of their lives. Despite the numerous benefits that are linked with providing infants with their mother's milk, a lot of mothers who have low birth weight (LBW) infants face challenges in expressing sufficient quantities of milk due to factors such as illness, stress, immaturity of mammary secretory cells, and other issues related to premature birth [2]. Donor human milk (DHM) is the prescribed alternative when the mother's milk is unavailable or insufficient. This suggestion aligns with commendations from the European Society for Paediatric Gastroenterology, Hepatology, and Nutrition, the American Academy of Pediatrics, and the WHO [3]. According to the 2018 recommendations from WHO or UNICEF, the emphasis was placed on providing DHM to infants who needed supplements or could not consume their mother's milk. This is particularly crucial for LBW infants, including those with very low birth weight (VLBW) and other vulnerable infants [4]. DHM refers to milk expressed and willingly contributed by lactating women who are not the biological mothers of the intended recipients [5]. DHM is beneficial because it enhances feeding tolerance and reduces the likelihood of necrotizing enterocolitis, bronchopulmonary dysplasia, and late-onset sepsis [6].

Since LBW is known to be a significant risk factor for both infant mortality and morbidity, it has grown to be a significant global public health issue [7]. In 2020, the number of babies born with LBW was 19.8 million, accounting for approximately 14.7% of all the new births taken worldwide for that year. This rate was noticeably higher in Southern Asia, reaching 24.4% [8]. The Academy of Breastfeeding Medicine advises selecting DHM as the primary supplement, giving it precedence over the formula. This emphasis is particularly notable in cases where supplementation becomes necessary, such as dehydration, hypoglycemia, and hyperbilirubinemia [9]. Over the recent decades, technological advancements in medical treatments have significantly enhanced the chances of survival of infants with VLBW. Nevertheless, despite these progressions, there has not been a proportional reduction in the associated morbidity linked to VLBW [10].

The utilization of donor milk in hospitals is influenced by several factors at both institutional and individual levels. The factors include the absence of standardized policies and insufficient staff training on its use. Additionally, the perspectives and knowledge of the staff members and parents regarding the positive side, i.e., health benefits and safety of donor milk, play a significant role in shaping its adoption [11]. Establishing the acceptability of human milk banking involves validating its value and addressing community beliefs and misconceptions. This can be achieved by offering comprehensive information and increasing awareness about the crucial role of DHM. This strategy is designed to enhance community conviction and promote acknowledgment of breast milk donation and the use of DHM [12]. This systematic review aims to provide insights into the importance of DHM in improving the outcomes in premature infants.

## Review

Methodology

This review explores the vital role DHM plays in the health outcomes of premature infants. A systematic literature search was carried out using PubMed and Google Scholar databases in conjunction with reliable sources such as the websites of UNICEF and WHO. The search strategy used was ((((((((((((infant) OR (newborn)) OR (neonate)) AND (low birth weight)) OR (preterm)) OR (early birth)) OR (premature)) AND (donor human milk)) OR (donor breast milk)) OR (donated mother milk)) OR (donor milk)) AND (milk bank)) OR (lactation management[MeSH Terms]), and filters were as follows: free full text in the last five years.

**Figure 1:**
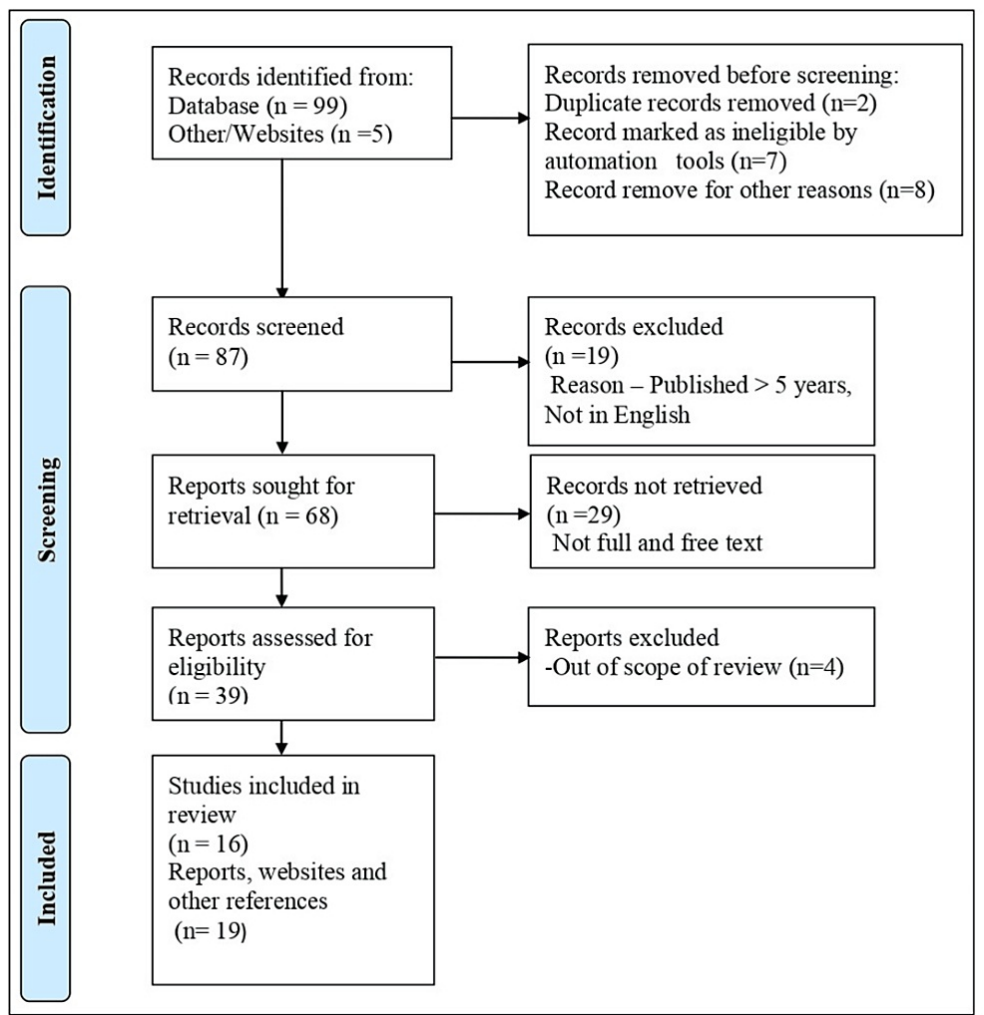
The selection process used in this study. Adopted from the Preferred Reporting Items for Systematic Reviews and Meta-Analyses (PRISMA).

We searched for further information using terms such as "lactation management," "human milk bank," "low birth weight," "immunity," and "preterm." Articles available in the English language and published in the previous five years, as well as free full-text and open access articles were included in this study. A total of 104 articles were searched, of which 35 were included after identification; filters were applied, eligibility was checked, and references that were out of scope, limited rigor, or had insufficient knowledge were excluded. Figure 1 shows the selection process used in the study.

Discussion

Donor Human Milk

DHM is obtained from donors and has undergone extensive screening, heat treatment, and microbiological screening before being supplied to patients in the community or medical facilities [13]. DHM represents human milk that has been pooled and pasteurized, meeting international standards for processing. Its application in premature infants has been associated with several beneficial health outcomes, including mitigation of feeding intolerance, necrotizing enterocolitis, late-onset sepsis, bronchopulmonary dysplasia, and various other conditions [14]. Substantial research suggests that human breast and donated human milk complement each other, proportionately improving child health and survival rates [15]. The complementing effect is particularly noticeable when all newborns are fed only breast milk. At birth, an infant's gastrointestinal tract and respiratory system possess low levels of antioxidant and anti-inflammatory properties associated with the immune system. In addition, it has poorly developed physical barriers, including tight junctions, a low gastrointestinal acidity (chemical barrier), a delayed T-cell response, and a decreased production of immunoglobulins, especially secretory immunoglobulin A (IgA). It has been shown that a healthy consumption of human milk influences the composition of gut bacteria, stimulates the gastrointestinal mucosa, and strengthens the developing child's immune system, probably because of the bioactivity in human milk [16]. Growth factors that show lower permeability of the intestinal lumen, which acts as a defense against ailments in a fragile gut, such as the epidermal growth factor seen in human milk, may support the development of small intestinal epithelium growth and gastro barrier protection [17].

Necrotizing enterocolitis: Necrotizing enterocolitis, which affects 5-12% of newborns with extreme LBW, is the leading cause of death in preterm neonates relating to gastrointestinal disorders. It can present with slow, subtle symptoms at first; some newborns show signs as early as feeding intolerance [18]. Indications of feeding intolerance include elevated gastric residual volume resulting in vomiting, abdominal distention, absence of stool, and more frequent apnea episodes [19]. The antitoxin activity of breast milk IgA against the enterotoxins of *Vibrio* *cholerae* and *Escherichia coli *may be necessary in avoiding infantile diarrhea [20]. In contrast to premature infants who were fed formulated milk, premature infants who were fed human milk have a lesser prevalence of necrotizing enterocolitis. Mother's milk contains bioactive substances, which consist of both bactericidal and immune-regulating properties that can protect against sepsis. Human milk has antibacterial properties that resist *Staphylococcus aureus*, *Candida *species, and *Escherichia coli* growth. It is recognized that DHM can be used in place of mother milk. Human milk has probiotics, prebiotics, l-arginine, glutamine, growth factors, oligosaccharides, and lactoferrin. These substances can help colonize good intestinal bacteria, prevent the multiplication of infectious microorganisms, maintain intestinal mucosa integrity, and increase resistance. As a result, this reduces the dangers of sepsis and necrotizing enterocolitis [14]. To reduce the risk of transmitting pathogens from donor mothers to preterm infants, DHM is commonly pasteurized. It is proven that DHM can be used in place of mother milk. The absence of milk banks can lead to detrimental consequences for infants [21].

Late-onset sepsis: Sepsis manifesting 72 hours postpartum is referred to as late-onset neonatal sepsis. It affects around 10% of babies born preterm and is linked to long-term neurological development problems [22]. DHM includes various immunological components, including antibodies such as IgA, IgG, or IgM, white blood cells, and immune-regulating substances. These elements enhance the infant's immune system, providing passive immunity and protecting against bacterial pathogens that could potentially cause sepsis [16]. Moreover, it promotes the development of a resilient gut barrier that helps in averting intestinal permeability. Consequently, this lowers the likelihood of bacteria moving from the gut to the bloodstream, decreasing systemic infections, including sepsis [23].

Bronchopulmonary dysplasia: Bronchopulmonary dysplasia is the predominant complication that arises from extremely premature birth. The affected infants show abnormal or impaired lung development, which may cause permanent changes in the function of the lungs and heart. One of the most critical components of its treatment and prevention is adequate nutritional support. The protective benefits of DHM against the development of bronchopulmonary dysplasia have been highlighted by several studies [24]. Preterm infants who receive human milk, whether from their biological mothers or through donors, may experience improved feeding tolerance compared to those formulated milk. This enhanced feeding tolerance can positively impact overall health and alleviate stress on the respiratory system [25]. Antibodies and other protective elements found in DHM prevent infections and also reduce the risk of lung inflammation and damage [20]. The well-balanced combination of proteins, fats, and carbohydrates in human milk has the potential to promote the growth and steady progress of premature infants [26].

Why Human Milk Over Formula Milk?

The infant's developing defense mechanism and shield against infections depend on a variety of bioactive substances and immune factors found in human milk, such as microRNAs, antibodies, immunoglobulins, lactoferrin, lysozyme, growth factors, antimicrobial peptides, white blood cells, and human milk oligosaccharides [27]. In a comparison between infants fed DHM and those fed their mother's milk, the latter had lower levels of Clostridiaceae and higher amounts of bifidobacteria. However, when it comes to the metabolic and functional traits of the microbiota, which differ significantly from those of newborns given formula, there is no discernible difference between the mother's milk and DHM [15].

DHM's advantages might result from less exposure to formula, which can exacerbate inflammation and intestinal permeability [17]. Formula feeds are expensive and may also result in dyspepsia. Higher energy and protein levels can be obtained from formula prepared from cow's milk. Still, human milk lacks many beneficial ingredients, such as the "personalized" ingredients exclusive to the mother's milk. Additionally, a formula may exacerbate inflammation and raise the possibility of necrotizing enterocolitis [28].

Breastfeeding Practices and Infant/Child Mortality Rates in India

LBW is defined as birth weight below 2,500 grams, and it is a significant public health problem in India as well as throughout the world. Infants weighing less than 1,500 grams are considered as VLBW, and it is associated with significant morbidity and mortality rates [29]. Even though there is a global decline in the child mortality rate, India's infant mortality rate remains higher than 30 per 1,000 live births. Lack of access to healthcare, female illiteracy, and ignorance of the dietary requirements of newborns and premature infants are the three main factors contributing to childhood malnutrition. Because of their medical conditions, these infants are often fed poorly and have problems in terms of longevity and cognitive development. The Indian state and union territory has a thorough report on population, health, and nutrition in the National Family Health Survey-5 (2019-21). According to this, the rate of children (below 3) who breastfed within an hour of birth is 41.8%, whereas 63.7% of infants younger than six months are exclusively breastfed. The rates of under-five mortality, infant MORTALITY, and neonatal mortality are reported to be 24.9%, 35.2%, and 41.9%, respectively, for every 1,000 live births [30]. Table 1 displays India's infant and child mortality rate and breastfeeding practices.

**Table 1:** Breastfeeding practices and infant/child mortality rates in India; NFHS-5 (2019-21). NFHS, National Family Health Survey

Sr. No.	Indicators	NFHS-5 (2019-21)			NFHS-4 (2015-16)
		Urban	Rural	Total	Total
Breastfeeding practices					
1.	Children who are breastfed within one hour of birth; below three years (%)	44.7	40.7	41.8	41.6
2.	Children exclusively breastfed; below six months (%)	59.6	65.1	63.7	54.9
Infant and Child death rate (per 1,000 live births)					
3.	Neonatal mortality rate (%)	18.0	27.5	24.9	29.5
4.	Infant mortality rate (%)	26.6	38.4	35.2	40.7
5.	Under-five mortality rate (%)	31.5	45.7	41.9	49.7

Human Milk Banking Process

**Figure 2:**
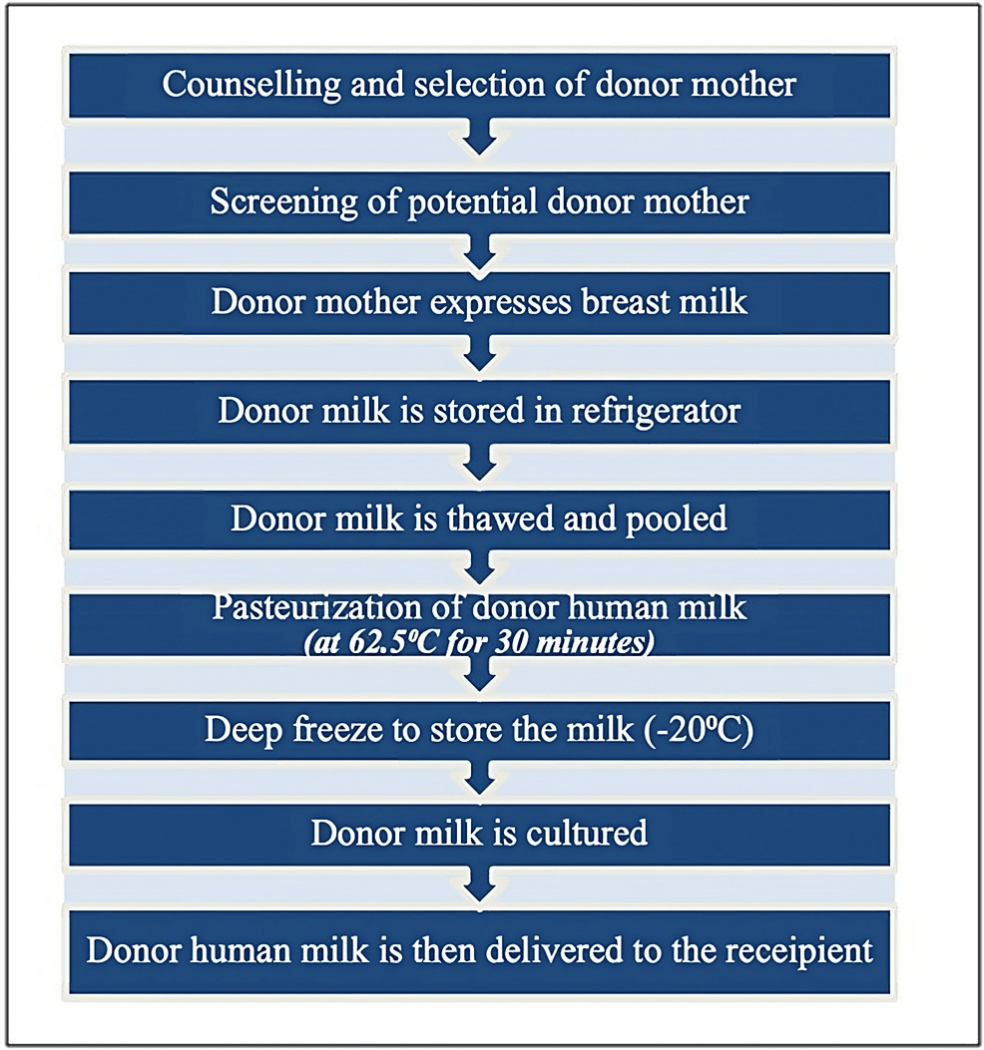
A flow chart illustrating the human milk banking process.

In 2019, an international expert gathering, co-sponsored by WHO and the University of Zurich, examined human milk banks' setup, operation, and supervision. The meeting underscored the need and consequences of handling the utilization of DHM in a way that gives precedence to safeguarding, promoting, and enhancing mother's milk whenever feasible [4]. Milk banks typically follow standardized procedures for gathering and handling donated milk. The milk bank provides guidelines about proper techniques for cleaning and pumping breasts to donors. It is standard practice to combine milk from different pumping sessions, and the collected milk is kept in containers provided by the bank. Each donor's name, expression time, and date must be properly labelled on each container [31,32]. A thorough screening procedure that involves an interview, a serological test, and medical clearance is required of all donors. Testing for hepatitis B and C, HIV, and the human T-cell leukemia virus are all included in serology. Once a mother is accepted as a donor, she receives training on the safe collection and storage of her milk. Subsequently, the milk is refrigerated, stored, and transported to the milk bank. After the milk has thawed, a bacterial culture is obtained. The milk is again cultured after being pasteurized for 30 minutes at 62.5°C in an industrial pasteurizer. As the final culture results are awaited, the milk is frozen again. The milk is delivered, thawed, and administered as needed once the milk bank requests human milk (Figure 2) [31].

Human Milk Bank Establishment in India 

Mother Milk Bank was founded under the name "Jeevan-Dhara" and was established in Udaipur in 2015 by an NGO with ties to the government. The first community milk bank, "Divya Mother Milk Bank," was founded in Udaipur as a private institution. Compared to other states, Rajasthan currently has the highest number of milk banks, roughly around 19, called Aanchal Mother Milk Banks [33]. Notably, in five years of bank operation, 13% of all donations came from mothers who delivered babies less than 500 grams, roughly 40% of this number referring to women who gave birth at exceptionally low gestational ages of less than 25 weeks. The composition data validates the exceptional value of human milk provided by mothers of premature newborns, making it a genuine biological jewel [15].

**Figure 3:**
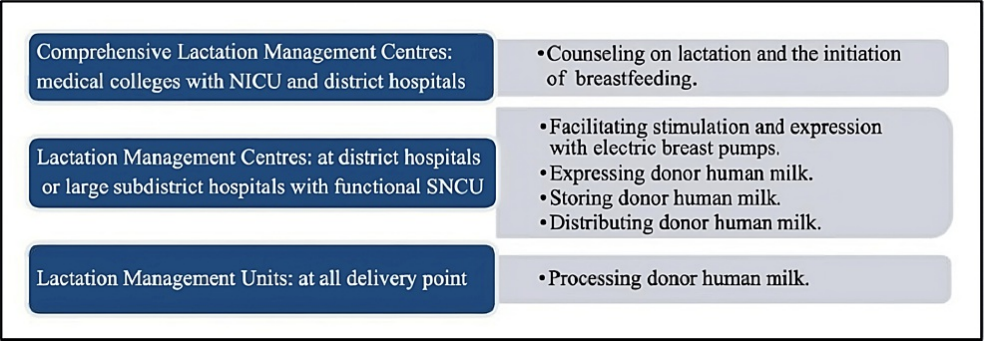
The roles and levels of centers that manage lactation, located in Indian facilities. NICU, neonatal intensive care unit; SNCU, special newborn care unit

In India, the establishment of human milk banks follows a structured framework known as the Lactation Management Centre (LMC), which operates across three distinct levels. Lactation Management units are located at all delivery sites; LMCs collaborate with Special Newborn Care Units at the district level, and Comprehensive LMCs are established to collaborate with medical colleges at the tertiary level. The National Guidelines on Lactation Management Centres in Public Health Facilities indicate the presence of approximately 80 operational milk banks nationwide (Figure 3) [34].

All hospitalized mothers can receive comprehensive lactation support and management from the Comprehensive LMC in a medical facility. It offers facilities for collecting, screening, filtering, storing, and distributing donated human milk to infants who receive their mother's milk. Furthermore, the Comprehensive LMC makes it easier for a woman to express and store breast milk for her child to consume [5].

The LMC is housed within a medical facility and was created to provide lactation support to all mothers who are patients there. Its primary purpose is to gather, store, and distribute a mother's breast milk so that her child can be fed [5].

The Lactation Support Unit is located at delivery points such as primary health centers, community health centers, and sub-district hospitals. Its purpose is to provide lactation support to all mothers receiving care at these healthcare institutions [5].

Problems Relating to Donating Human Milk

Individual barrier: The most often mentioned obstacles were mothers' ignorance of the donation process, their lack of time because of everyday chores, the physical and mental strain of pumping, freezing, or storing milk, and the expense and travel time to the milk bank [35].

Social barrier: Women face barriers because societal taboos prohibit them from giving or receiving. The fact that giving milk requires time puts a stop to their willingness to do so. Religious customs were the primary cause of the reluctance to accept donated DHM for feeding and to donate breast milk. The primary obstacle to providing mothers with counselling was cultural reluctance to view milk banking as unethical. For various reasons, including the possibility of infection, the loss of their child's attachment, concerns related to safety, and the loss of milk nutrients, some mothers declined to take the given milk [33].

Systemic barrier: The health system-related barriers to breast milk donation were identified as follows: inadequate infrastructure; the improper acquiring and storage of breast milk; the absence of trained staff; testing in laboratories; equipment upkeep; and the potential for contaminated milk; particularly during thawing. These issues must be taken care of by ensuring enough safety measures. Hospitals discouraging donation due to infection risk were also identified [35].

Recommendation

Human milk banking should be incorporated into programs encouraging breastfeeding, highlighting lactation in mothers and only using DHM when required [32]. Promote the creation and advancement of various plans of action that ease the use of DHM within healthcare institutions. Urge healthcare providers to inform parents about the accessibility and advantages of DHM, especially in neonatal intensive care units and similar settings catering to preterm infants in need of extra assistance.

Training should be provided to the staff to address any potential cultural barriers that might affect the relationships between donors and recipients. Social Behavior Communication Change intervention should target specific groups to remove obstacles and eliminate common misconceptions about milk banking procedures among communities.

We can address worldwide inequities in accessing DHM by focusing on the expansion and long-term sustainability of human milk banking in poor resource areas [26]. Public policies must be created to improve access to DHM and raise public knowledge of its advantages, particularly among groups whose breastfeeding rates are low [32].

Provide an ethical framework to direct national laws regarding the proper procurement and application of DHM. The framework would be based on the ideas of justice and equity, and it would include safeguards for both milk givers and recipients as well as a reverence for autonomy and human rights. Before proceeding, the donor and the recipients should provide informed consent. Table 2 shows the summary of the included studies.

**Table 2:** List of studies included in the review. DHM, donor human milk; LBW, low birth weight; IgA, immunoglobulin A; VLBW, very low birth weight

Author	Year	Finding
Bramer et al. [1]	2021	DHM can lessen the incidence and severity of bronchopulmonary dysplasia and necrotizing enterocolitis in preterm infants.
Unger et al. [2]	2014	Donor milk banks handle large volumes of products and require strict protocols to prevent contamination, misidentification, or infection; they require a significant financial outlay.
Piemontese et al. [6]	2019	One benefit of using pooled milk is that it contains donor milk from various lactation stages.
Singh et al. [7]	2023	According to the WHO and UNICEF, low socioeconomic status, poor diet, infections, and physical labor during pregnancy account for 96% of LBW cases.
Sen et al. [9]	2018	Banked donor milk is a potentially finite resource and previously saved for premature or ill newborns in the neonatal intensive care unit.
Yang et al. [10]	2020	While pasteurization removes immune cells from human milk, it does not destroy biological activity completely; many bioactive ingredients, such as growth factors and cytokines, are still present.
Kimani‐Murage et al. [12]	2019	DHM would reduce morbidity, particularly from the adverse effects of delayed breastfeeding and baby formula allergies.
Shenker et al. [13]	2023	The possibility that mothers may become less motivated to establish the milk supply if DHM is readily available has been one of the main arguments made against its use.
Young et al. [14]	2020	During the first year of lactation, milk IgA gradually drops; DHM total IgA was not correlated with milk postpartum age.
Hoban et al. [17]	2020	The advantages linked to the DHM era in this research might simply result from a reduction in formula exposure, which has been known to raise intestinal permeability and inflammation.
Costa et al. [19]	2018	In comparison to donor breast milk, formula feeding may boost short-term growth rates in preterm or birth-weight infants, but it also doubles the risk of necrotizing enterocolitis development, according to a Cochrane review by Quigley and McGuire.
Arboleya et al. [23]	2020	A distinct gut microbiota profile is observed in premature babies fed DHM as compared to babies breastfed by their mothers.
Villamor-Martínez et al. [24]	2018	In very preterm/VLBW infants, DHM may protect against bronchopulmonary dysplasia. Pasteurization, on the other hand, seems to lessen the advantageous qualities.
Kumbhare et al. [28]	2022	The gut microbiome development of VLBW infants was found to be significantly influenced by the source of human milk (own mother versus donor milk), while the type of fortifier (human versus bovine) had a negligible effect.
Mantri et al. [33]	2021	Findings from Ethiopia are consistent with this study, which found that some mothers were hesitant to give their infants donated human milk because of the possibility of disease transmission.
Mathias et al. [35]	2023	According to many studies, one of the biggest obstacles to milk donation is people's ignorance of milk banking. These results align with those obtained by Wambach et al.

## Conclusions

Exclusively breastfeeding for the first six months of life is the most nutritious food source. It is well-known that human milk helps prevent various infant diseases and is beneficial to the health of premature infants. In situations where nursing presents difficulties because of illnesses or for any other reason, DHM may be used. For premature babies, DHM is crucial because it is a transitional food source for effective breastfeeding. Although it is handled and processed by human milk banks, its qualities help to prevent postnatal growth deficits and offer health benefits. In addition, DHM lessens the need for formula, a known risk factor for bronchopulmonary dysplasia, late-onset sepsis, and necrotizing enterocolitis. Milk must be pasteurized using a high-quality holder to preserve essential nutrients and strengthen newborns' immune systems. Particularly for premature infants, human milk banking is an absolute necessity. Inequities in the prevention of diseases with high death rates may result from a lack of access to socio-health infrastructures, such as milk banks. To sum up, DHM enhances susceptible infants' overall health and well-being by helping them meet their nutritional needs.
